# A Validated Densitometric Method for Analysis of Atorvastatin Calcium and Metoprolol Tartarate as Bulk Drugs and In Combined Capsule Dosage Forms

**DOI:** 10.4103/0975-1483.76420

**Published:** 2011

**Authors:** SM Patole, AS Khodke, LV Potale, MC Damle

**Affiliations:** *Department of Quality Assurance, A.I.S.S.M.S. College of Pharmacy, Kennedy Road, Near R.T.O., Pune - 411 001*; 1 *Assistant Professor, Department of Pharmaceutical Chemistry, A.I.S.S.M.S. College of Pharmacy, Kennedy Road, Near R.T.O., Pune - 411 001, India*

**Keywords:** Atorvastatin calcium, high performance thin layer chromatography, metoprolol tartarate, validation

## Abstract

A simple, accurate and precise high-performance thin-layer chromatographic method has been developed for the estimation of Atorvastatin Calcium and Metoprolol Tartarate simultaneously from a capsule dosage form. The method employed Silica gel 60F _254s_precoated plates as stationary phase and a mixture of Chloroform: Methanol: Glacial acetic acid (dil.) :: (9:1.5:0.2 ml %v/v) as mobile phase. Densitometric scanning was performed at 220 nm using Camag TLC scanner 3. The method was linear in the drug concentrations’ range of 500 to 2500 ng/spot for Atorvastatin Calcium, also for Metoprolol Tartarate with correlation coefficient of 0.984 for Atorvastatin Calcium and 0.995 for Metoprolol Tartarate respectively. The retention factor for Atorvastatin Calcium was 0.45 ± 0.04 and for Metoprolol Tartarate was 0.25 ± 0.02. The method was validated as per ICH (International Conference on Harmonisation) Guidelines, proving its utility in estimation of Atorvastatin Calcium and Metoprolol Tartarate in combined dosage form.

## INTRODUCTION

Atorvastatin Calcium is chemically known as (βR, 8R)-2-(4-fluorophenyl)-α, δ-dihydroxy-5-(1-methylethyl)-3-phenyl-4-[(phenylamino) carbonyl] 1H-pyrrole-1-heptanoic acid trihydrate.[1] Metoprolol Tartarate is chemically known as(RS)-1-isopropylamino-3-p-(2-methoxyethyl)phenoxypropan-2-ol(2R,3R)-tartrate.[1] Atorvastatin Calcium is a member of the drug class known as statins, used for lowering blood cholesterol. It also stabilizes plaque and prevents strokes through anti-inflammatory and other mechanisms. Metoprolol Tartarate is an antihypertensive drug, It is a selective β1 receptor blocker used in the treatment of several diseases of the cardiovascular system, especially hypertension. Literature survey revealed that assay for Atorvastatin Calcium as bulk and its dosage form are official in Indian Pharmacopoeia 2007[2] and for Metoprolol Tartarate is official in British Pharmacopoeia 2007.[3] RP-HPLC method has been reported for the estimation of Atorvastatin Calcium Fenofibrate.[4] Analytical methods reported for the estimation of Atorvastatin Calcium are HPLC,[5–8] Stability indicating High Performance Liquid Chromatography (HPLC)[9,10] High Performance Thin Layer Chromatography (HPTLC)[11,12] and Spectrophotometry.[13,14] Analytical methods reported for the estimation of Metoprolol Tartarate are Spectrophotometry[15] and HPLC.[16] Referring to the literature survey, there is no published HPTLC method for this combination. The present paper describes a simple, accurate and precise method for simultaneous estimation of Metoprolol Tartarate and Atorvastatin Calcium in combined capsule dosage form. The proposed method is optimized and validated as per the International Conference on Harmonization (ICH) guidelines.

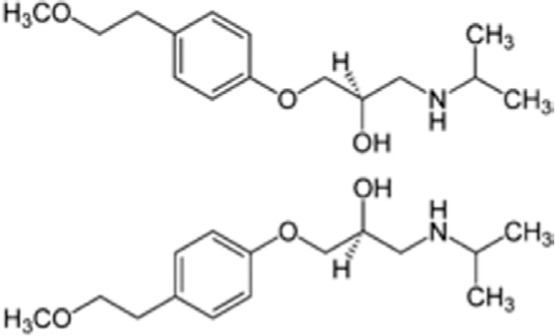



Metoprolol Tartarate structure

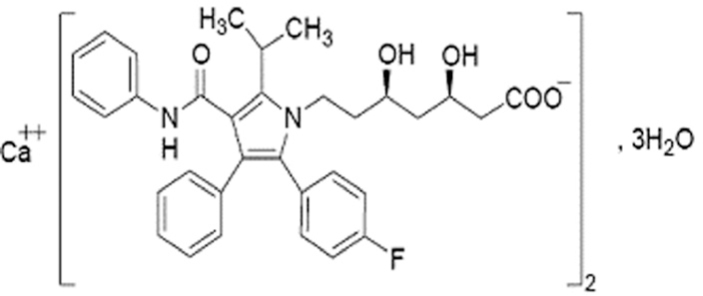



Atorvastatin Calcium structure

## MATERIALS AND METHODS

Working standard of Atorvastatin Calcium and Metoprolol Tartarate was procured from Shreya Pharmaceuticals, Aurangabad, India as gift samples. Marketed formulation Betaone (Metoprolol Tartarate-25 mg and Atorvastatin Calcium-10 mg/capsule) was purchased from local market.

All other reagents used for experimentation were of analytical reagent (AR) grade. Chemicals used for this experiment were Acetonitrile, Methanol, Chloroform, Sodium hydroxide, Hydrochloric acid, and Hydrogen Peroxide.

### Instruments

HPTLC system (Camag, Muttenz, Switzerland) comprising Linomat 5 sample applicator, twin-trough development chamber and TLC Scanner 3 with WinCATS evaluation ATS software (Version 1.4.3) was used in the studies. Electronic balance (Make *SHIMADZU* Model *AY-120*) was used for weighing purpose.

## PROCEDURE

### Preparation of standard stock solution

Ten mg each of Atorvastatin Calcium and Metoprolol Tartarate were weighed and transferred to 10-ml volumetric flask. Methanol (AR) was added to dissolve the drug and final volume was made with the same solvent to obtain a concentration 1000 µg/ml of each drug. Appropriate amount of stock solutions were diluted with methanol to obtain a concentration of 100 µg/ml of Atorvastatin Calcium and Metoprolol Tartarate.

### Method development

Initially, trials were taken on TLC plates precoated with Silica gel 60F _254_. Each standard was applied as bands on TLC plates in five replicates. Plates were developed by linear ascending development using neat solvents like methanol, chloroform, acetic acid, acetone, etc. with chamber saturation. Based on the results of these initial chromatograms binary and ternary mixtures of solvents were tried to achieve optimum resolution with Atorvastatin Calcium and Metoprolol Tartarate respectively. Trials on TLC plates precoated with Silica gel 60F _254_showed movement of both drugs at nearly the same R _f_. After several trials, mixture of Chloroform: Methanol: Glacial acetic acid (dil.):: (9:1.5:0.2ml v/v) was chosen as the mobile phase for analysis which showed good separation for these two drugs. The linearity of the method was determined at five concentration levels ranging from 500 to 2500 ng/spot for Atorvastatin Calcium and the same for Metoprolol Tartarate.

### Procedure for analysis of capsule formulation

**Figure 1:**
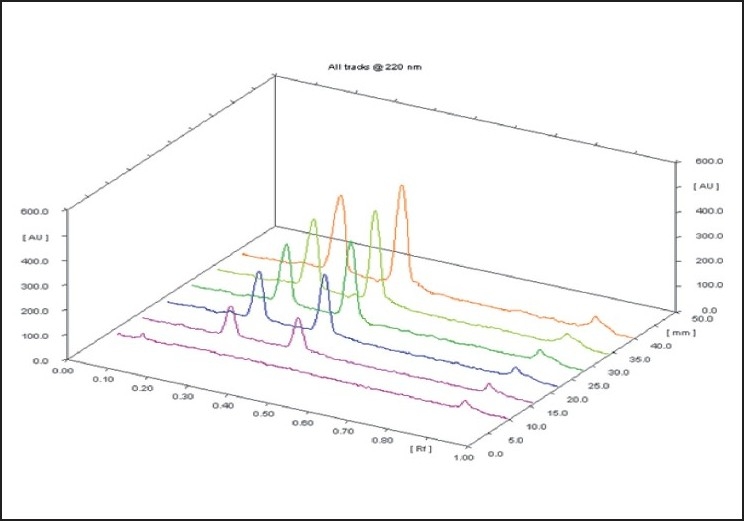
Representative densitogram (linearity) of atorvastatin calcium and metoprolol tartarate

Four capsules were weighed accurately and powder content was removed. Powder quantities equivalent to 10 mg of Atorvastatin Calcium and 25 mg of Metoprolol Tartarate were weighed separately and transferred to 25-ml volumetric flask and volume was made up. The resultant solution was further sonicated for 10 min. The volume was then made up to 10 ml with same solvent. Each solution was then filtered through whatmann filter paper no.41. From the filtrate, appropriate volumes were spotted to obtain final concentration of 320 ng/spot for Atorvastatin Calcium and 800 ng/spot for Metoprolol Tartarate. Spotting was done in the form of bands and plate was developed up to a distance of 90 mm, using the mobile phase Chloroform: Methanol: Glacial acetic acid (dil.) :: (9:1.5:0.2ml v/v) in normal conditions of temperature and humidity. The peak areas of the spots were measured at 220 nm where both the drugs showed considerable absorbances [Figure 1] and concentrations in the samples were determined from the respective calibration curves. The amount of each drug present per capsule was calculated.

### Method Validation

As per the ICH guidelines, the method validation parameters checked were linearity and range, accuracy, precision, specificity, limit of detection, limit of quantitation and robustness.

#### Linearity

Different concentrations of Atorvastatin Calcium (500 ng to 2500 ng/band) and Metoprolol Tartarate (500 to 2500 ng/band) were applied on TLC plate and densitograms were developed. The data of peak area vs. drug concentration was obtained by linear least-square regression analysis.

#### Precision

Inter-day and intra-day precision was evaluated by analyzing sample preparations obtained from homogenous sample, six times and % RSD (Relative Standard Deviation) value obtained was calculated to determine any intra-day and inter-day variation.

#### Accuracy

To check accuracy of the method, recovery studies were carried out by addition of standard drug solution to pre-analyzed sample solution at three different levels 80, 100 and 120%. Mean percentage recovery was determined.

#### Limit of detection and limit of quantification

The limit of detection (LOD) and limit of quantification (LOQ) were obtained by calculations, using the standard formula as per the ICH guidelines,

$$\mathrm{LOD}\;=\;\frac{3.3\;\sigma }{S}\;\;\;\;\;\;\;\mathrm{LOD}\;=\;\frac{10\;\sigma }{S}\;$$

where σ is Standard deviation of the response and S is slope of the calibration curve.

#### Specificity

The specificity of the method was ascertained by peak purity studies. Purity of the drug peaks was ascertained by analyzing the spectrum at peak start, max position and at peak end. The peak purity was determined by win CATS software.

## RESULTS AND DISCUSSION

### Linearity and Range

**Figure 2:**
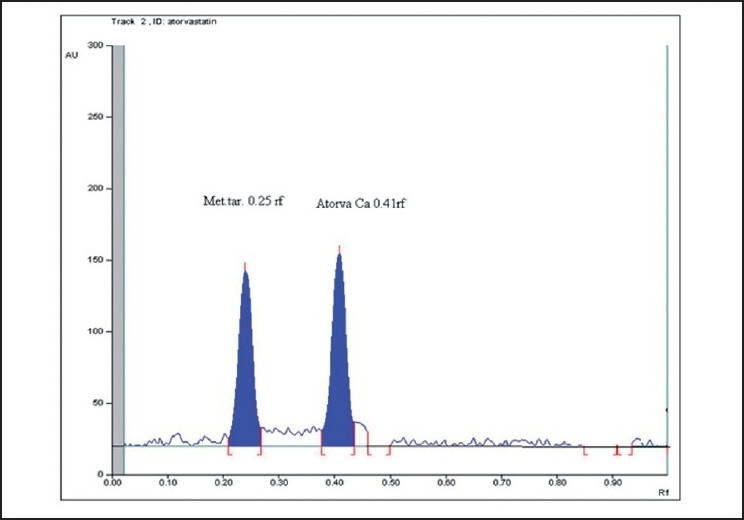
Representative densitogram of atorvastatin calcium with Rf 0.45 and metoprolol tartarate with Rf 0.25

**Figure 3:**
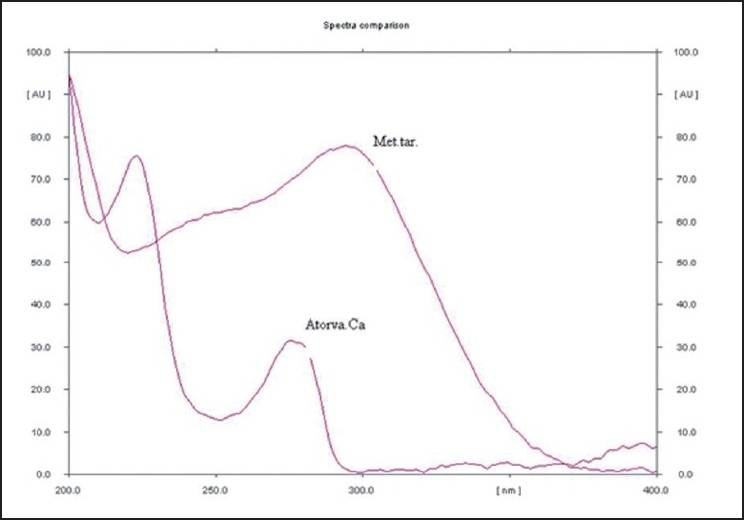
Representative spectral comparison of atorvastatin calcium and metoprolol tartarate

Linearity of the method was found in the range of 500 to 2500 ng/spot for Atorvastatin Calcium and 500 to 2500 ng/spot for Metoprolol Tartarate Figure 2. Rf for Atorvastatin Calcium and Metoprolol Tartarate were 0.45 and 0.25 respectively Figure 3. The linearity is indicated by regression equation. The linear regression equations obtained are:

For Atorvastatin Calcium y = 2.209× + 556.3 (r^2^ = 0.9991)

For Metoprolol Tartarate y = 1.853× + 250.9 (r^2^ = 0.9950)

### Accuracy

Excellent recoveries were obtained at each level of added concentration. The results obtained (n = 3 for each 80%, 100%, 120% level) indicated the recovery was within the range of 100 ± 2% which indicates accuracy of the method. Results are shown in Table 1

**Table 1:** Recovery studies of Atorvastatin Calcium and Metoprolol Tartarate

Level	Conc	(ng/spot)	Area	Mean	Recovered	%
	Std.	Sample			Conc.	Recovery
80	700	+ 620	3439	3449	1309	99.20
			3447			
			3459			
100	700	+ 700	3548	3561	1360.2	97.14
			3554			
			3567			
120	700	+ 780	3829	3826	1489	100.62
			3821			
			3825			

Level	Conc	(ng/spot)	Area	Mean	Recovered	%
	Std.	Sample			Conc.	Recovery

80	1000	+ 800	3549	3555	1783	99.06
			3559			
			3555			
100	1000	+ 1000	3952	3930	1985.4	99.27
			3902			
			3936			
120	1000	+ 1200	4314	4322	2197	99.86
			4329			
			4324			

### Precision

In the intra-day studies, three different concentrations of the mixed standard were analyzed in a day and percentage RSD was calculated and was found to be less than 1.5%. In the inter-day variation studies, three different concentrations of the mixed standard were analyzed on three consecutive days and percentage RSD was calculated which was found to be less than 1.5%. The results of the inter-day and intra-day studies are shown in Table 2. The data obtained indicates that the developed HPTLC method is precise.

**Table 2:** Validation parameters for Atorvastatin Calcium and Metoprolol Tartarate

Parameters	Atorvastatin calcium	Metoprolol tartarate
Range	500-2500 ng/spot	500-2500 ng/spot
% RSD (n=3)		
Intra-day precision	0.86	0.72
Inter-day precision	0.84	0.40
Accuracy (recovery)	Within 98-102%	Within 98-102%
Robustness	Robust	Robust
LOD (ng/spot)	46.46	23.52
LOQ (ng/spot)	151.30	71.28
Specificity	Peak purity > 0.9950	Peak purity > 0.9950

LOQ^a^: Limit of detection, LOD^b^: Limit of quantification

### Limit of Detection and Limit of Quantification

The LOD and LOQ values are shown in Table 2.

### Robustness

The robustness of an analytical procedure is a measure of its capacity to remain unaffected by small but deliberate variations in procedure. Slight and deliberate changes were made to the following parameters like changing the mobile phase ratio and chamber size, and the effect on the Rf values and peak areas were noted. The method was found to be robust since the monitored parameters were not significantly affected.

### Specificity

Win Cats software afforded automatic calculation of the peaks’ purity by comparing the UV spectra, at the start, max and tail of the peak of each response. The correlation value r > 0.99 confirmed peak purity indicating that this HPTLC method is able to resolve and accurately measure two drugs in the presence of the matrix components. The peak purity values were found to be more than 0.9950 which show that the method is specific for the two drugs.

## DISCUSSION

The method was found to be simple, fast and precise for the estimation of Atorvastatin Calcium and Metoprolol Tartarate simultaneously from a capsule dosage form. The accuracy of the method was found to be good. The method is also specific as confirmed by peak purity studies.

## CONCLUSION

The proposed HPTLC method for the simultaneous estimation of Atorvastatin Calcium and Metoprolol Taratrate in combined dosage forms was found to be sensitive, accurate, precise, simple and rapid. Hence the present HPTLC method may be used for routine analysis of these drugs and their formulations.
